# Exploring AI in the Multigenerational Workplace: Opportunities, Challenges and Best Practices

**DOI:** 10.1093/geroni/igaf122.1368

**Published:** 2025-12-31

**Authors:** Samantha Brady, Lauren Cerino

**Affiliations:** Massachusetts Institute of Technology, Cambridge, Massachusetts, United States; Massachusetts Institute of Technology, Cambridge, Massachusetts, United States

## Abstract

Artificial Intelligence (AI) is rapidly changing the nature of work across nearly all industries. With the potential to enhance efficiency, decision-making, and innovation, many workplaces are adopting a range of AI tools and solutions. In light of the rapid expansion of AI in the workplace as well as the aging of the U.S. workforce, it is imperative to understand the ways in which AI can support older workers while also presenting new challenges for this growing segment of workers. To understand the opportunities and challenges AI presents for workers across generations, we conducted in-depth interviews with human resource managers and executives across a variety of industries in the U.S. Interviews highlighted potential benefits of AI for older workers including greater productivity, work quality improvements, and the ability to shift from administrative and logistical tasks to more human-centered work. Additionally, AI can assist with the documentation process necessary for knowledge transfer, especially in organizations with a large population of older workers. However, HR professionals also identified key challenges, particularly in multigenerational workplaces. These include training and education gaps, usability concerns, data security risks, and differing mental models of work that may complicate AI adoption. This session will examine both the opportunities and obstacles AI presents across generations and offer best practices for organizations seeking to integrate AI effectively. By considering the unique needs of older workers, organizations can create a more inclusive and adaptive workplace for the future.

